# Heart-focused anxiety in critically ill patients' relatives

**DOI:** 10.1186/cc13216

**Published:** 2014-03-17

**Authors:** Z Konstanti, M Gouva, G Nakos, V Koulouras

**Affiliations:** 1 University of loannina, Greece

## Introduction

A series of studies have shown an association between the ICU environment and symptoms of psychological distress in critically ill patients' relatives [1-3]. The aim of this study was to investigate the risk of cardiophobia and panic attack crisis on ICU patients'family members.

## Methods

In the period from March 2012 to July 2013, we studied 223 family members (81 men and 142 women, mean age 41.5 ± 11.0 years) of critically ill patients. These patients were admitted to the intensive care department with various medical and surgical conditions (147 patients, 103 men and 44 women, mean age 58.3 ± 18.6 years). We used a questionnaire in which were included: social-demographic characteristics of the patients' family environment, and the Cardiac Anxiety Questionnaire (CAQ). The results from the CAQ [4] were compared with those of the general population of Greece (GGP).

## Results

In comparison with the general Greek population, the relatives of critically ill patients demonstrate statistically significant higher scores (*P *< 0.001) on the scales of fear and anxiety in regards of thoracic and heart sensations (1.1 ± 0.9 vs. 0.9 ± 0.9 in GGP), avoidance of activities considered to reproduce cardiac symptoms (1.3 ± 1.0 vs. 1.1 ± 0.9 in GGP), heart-focused attention and self-monitoring of cardiac activity (0.9 ± 0.7 vs. 0.7 ± 0.6 in GGP), and at total CAQ score (1.1 ± 0.6 vs.0.9.± 0.6 in GGP). Analysis of variance among family members on heart- focused attention and self-monitoring of cardiac activity showed that hospitalization provoked heart-focused attention (that is, the main factor of perceived panic crisis risk). In accordance with the Bonferroni criterion, we found that patients' siblings demonstrated statistically significant difference compared with patients' children (*P *= 0.015), with the latter showing lower levels of heart-focused attention and selfmonitoring of cardiac activity. Further MANCOVA analysis showed a statistically significant correlation between age and cardiophobia, and also between education level and heart-focused anxiety (*P *= 0.041 and *P *= 0.044 respectively).

## Conclusion

The hospitalization of a patient in the ICU is considered to be a factor that is affecting the mental health of patients' relatives. Our results highlight a risk for psychologically induced symptoms on ICU relatives and underline the need for a psychosocial support system for them. The cardiophobia and self-monitoring of heart activity which inflicts the patients' relatives appears to be more intense in the siblings, parents and partners of patients.
